# Meth Mouth—A Growing Epidemic in Dentistry?

**DOI:** 10.3390/dj5040029

**Published:** 2017-10-30

**Authors:** Andreas Pabst, Juan Carlos Castillo-Duque, Axel Mayer, Marcus Klinghuber, Richard Werkmeister

**Affiliations:** Department of Oral- and Maxillofacial Surgery, Federal Armed Forces Hospital, Rübenacherstrasse 170, 56072 Koblenz, Germany; carloscastilloduque@bundeswehr.org (J.C.C.-D.); axelmayer@bundeswehr.org (A.M.); marcusklinghuber@bundeswehr.org (M.K.); rwerkmeister@bundeswehr.org (R.W.)

**Keywords:** methamphetamine, crystal, meth, meth mouth, jaw necrosis, MRONJ

## Abstract

In the past two decades, the synthetic style and fashion drug “crystal meth” (“crystal”, “meth”), chemically representing the crystalline form of the methamphetamine hydrochloride, has become more and more popular in the United States, in Eastern Europe, and just recently in Central and Western Europe. “Meth” is cheap, easy to synthesize and to market, and has an extremely high potential for abuse and dependence. As a strong sympathomimetic, “meth” has the potency to switch off hunger, fatigue and, pain while simultaneously increasing physical and mental performance. The most relevant side effects are heart and circulatory complaints, severe psychotic attacks, personality changes, and progressive neurodegeneration. Another effect is “meth mouth”, defined as serious tooth and oral health damage after long-standing “meth” abuse; this condition may become increasingly relevant in dentistry and oral- and maxillofacial surgery. There might be an association between general methamphetamine abuse and the development of osteonecrosis, similar to the medication-related osteonecrosis of the jaws (MRONJ). Several case reports concerning “meth” patients after tooth extractions or oral surgery have presented clinical pictures similar to MRONJ. This overview summarizes the most relevant aspect concerning “crystal meth” abuse and “meth mouth”.

## 1. Introduction

Methylamphetamine (methamphetamine, *N*-methyl-alpha-methyl-phenetyl-amine) was first synthesized in liquid form by Japanese chemists in 1893. In 1919, the pure form was crystallized and then patented for commercial distribution in 1921 [1,2]. In the 20s and 30s of the last century, the medical and paramedical use of methamphetamine started in Europe and spread rapidly around the globe. In Germany, Temmler Industries (Berlin) started to produce methamphetamine under the brand name “Pervitin” in 1938 [1]. 

In World War II, methamphetamine was widely used by all warring parties to keep the soldiers in permanent physical and mental efficiency. “Pervitin” was given to the German soldiers, “Philopon” to the Japanese, and “Methedrine” to the US and UK soldiers, especially to fighter pilots such as the Japanese Kamikaze, submarine crews, and special forces such as paratroops [1]. After World War II, the Federal Armed Forces of West Germany and the National People’s Army of East Germany also stored “Pervitin” until the end of the Cold War, and it was commercially available until the 80s of the 20th century [1].

The pharmacological mechanism of methamphetamine is based on the release and increased concentration of the neurotransmitters dopamine (DA) and noradrenaline (NA) in the central nervous system (CNS). Within the presynaptic cells, methamphetamine induces the release of DA and NA from the vesicles into the cytoplasm and eventually into the synaptic cleft. The effects of methamphetamine on NA release are much stronger than on DA release [2]. Calipari and Ferris reported that methamphetamine is further able to decrease DA and NA re-uptake from the synaptic cleft into the presynaptic cell by inhibiting DA and NA membrane transporters and by a reverse-transport of NA and DA into the synaptic cleft that is independent from an action-potential-caused vesicular release [3].

Methamphetamine stimulates the CNS and can significantly increase users’ physical and mental efficiency. It increases energy, perseverance, alertness, concentration, risk appetite, libido, and sexual desire. In addition to this, it induces mental euphoria and self-esteem and is able to efficiently suppress hunger, thirst, and the sensation of pain [2,4].

## 2. “Crystal Meth”

As a fashion and designer drug, “crystal meth” abuse originally started in California (United States) in the early 1990s and spread over the entire United States [4]. Chemically, it represents the bitter tasting, white crystalline salt of the methamphetamine hydrochloride ((*S*)-*N*-methyl-1-phenyl-propane-2-amine), which is a colorless, insoluble volatile oil [1]. “Crystal meth” came to Europe via the Czech Republic in the early 2000s, spread like wild fire across of all of Eastern Europe and then moved on to Central and Western Europe [1]. Currently, there are about 35 million users world-wide [2]. Further popular names for illegal methamphetamine products—besides “crystal meth”—are “crystal”, “meth”, “ice”, “crank”, “crypto” and “fire”. The crystalline form is most often smoked [1,4,5]. “Meth” is also often combined with other drugs: croak (with cocaine), shabu (with cocaine), and twisters (with crack) [4]. 

The main side effects of long-term chronic “meth” abuse include physical and mental dependence, increased blood pressure, severe cardiovascular diseases (e.g., heart attack, stroke, aneurysms), kidney failure, premature aging and physical decay, extreme weight loss, seizures, anxiety, nervousness, confusion, insomnia, paranoia, visual and auditory hallucinations, delusions, mood disturbances, anticholinergic syndrome, logorrhea, xerostomia, and much more [1,2,4]. “Meth” is also able to reduce the potency of antipsychotic drugs, such as antidepressants [4]. Its wide dissemination is due to, among other aspects, its low price, simple fabrication, ease of availability, and the quick, strong dependency.

## 3. “Meth Mouth” and Jaw Necrosis

**Figure 1:**
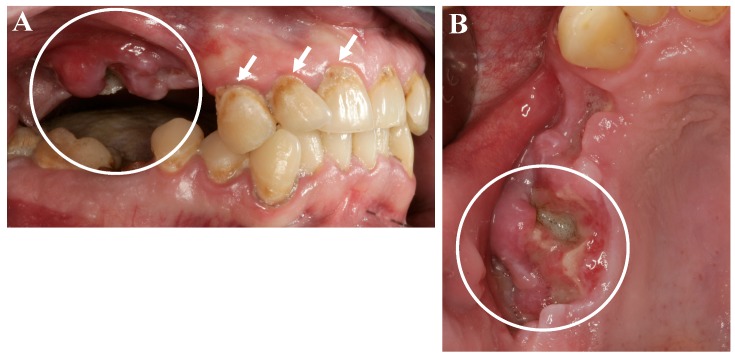
Oral and dental situation of a young patient after long years of methamphetamine abuse. (**A**) Characteristic cervical carious lesions (white arrows) and a severe wound healing disturbance with exposed jaw bone and a superinfection of the surrounding tissues of the maxilla (white circle), two weeks after tooth extraction *alio loco*; (**B**) View on the exposed bone of the maxilla (white circle) [9].

**Figure 2:**
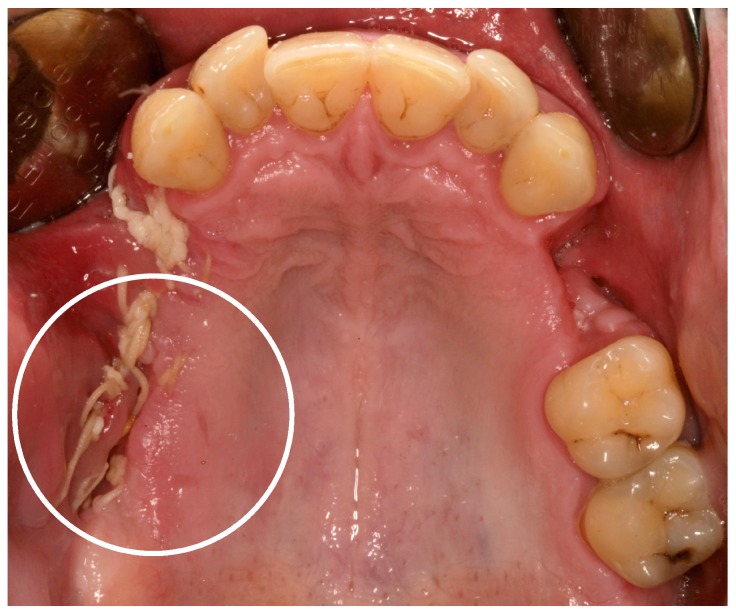
Twenty-one days postoperatively the wound bed still looked fragile and not “stage-related” (white circle). After the removal of the sutures, wound bed has immediately ruptured again [9].

As a further side effect, a condition known as “meth mouth” has been increasingly reported and is characterized by xerostomia, extensive carious lesions, enamel erosions, extensive teeth crunching, bruxism, muscle trismus, and lockjaw [1,2,6], see Figure 1 and Figure 2.

In recent years, case reports and studies on osteonecrosis after long-term methamphetamine abuse similar to the medication-related osteonecrosis of the jaws (MRONJ) have been published. Basin et al., reported cases of jaw necrosis in patients after desomorphin abuse in Russia, which has been confirmed by further studies [5,6,7,8]. Recently, Pabst and Werkmeister presented a case report of a young patient (male, 26 years) after four years of “meth” abuse and the development of an extended ONJ in the maxilla after tooth extractions, see Figure 1 and Figure 2 [9]. The patient had no further general diseases or allergies. His patient history was unobtrusive with exception of the “meth” abuse. A surgical wound revision of the affected areas was performed with general anesthesia, including a resection of necrotic bone and a saliva-proof wound closure under antibiotic treatment. The sutures were removed on day 21 postoperatively. To date, regular intraoral soft-tissue conditions can be observed. Further, restorative dentistry has been performed in private practice to restore the remaining teeth [9].

These investigations led to the assumption and the hypothesis that methamphetamine abuse might be—in addition to bisphosphonates (e.g., alendronate, zoledronate), anti-RANKL antibodies (denosumab), anti-VEGF antibodies (bevacizumab), tyrosinkinase inhibitors (sunitinib), estrogen receptor modulators (e.g., Raloxifen) and methotrexate—a further possible reason for MRONJ development [5,10,11,12,13,14,15,16,17,18].

This case report presents the case of a young patient with a years-long history of “meth” abuse and the resulting oral and dento-alveolar consequences, representing a special challenge in terms of therapy and aftercare. In addition, the most relevant literature on the topic is summarized and assessed.

## 4. Discussion

To date, there is no comprehensive, provable relationship between methamphetamine abuse and the development of “meth mouth”; more particularly the development of methamphetamine-induced osteonecrosis of the jaws has not been proven. Existing knowledge is based on individual case reports and a few small-scale studies [2]. Therefore, the exact pathophysiological mechanisms are the purpose of current research.

Consequently, Rommel et al., analyzed saliva flow rate, saliva production, and saliva pH values. Further, bruxism symptoms (tooth attrition, dentine exposure and enamel cracks) and muscle trismus in 100 chronic methamphetamine abusers and controls were evaluated. The findings demonstrated that methamphetamine abuse can induce extensive xerostomia via a reduced saliva flow rate and production, decreased saliva pH-values, and extensive bruxism. This might induce the general clinical picture of “meth mouth” by inducing extensive carious lesions and teeth crunching [2]. Ravenel et al., performed a similar study with a reduced number of patients (28 meth abusers and 16 controls). The authors could not find differences in the salivary flow rate between methamphetamine abusers and the controls. Lower saliva pH values and buffer capacity were confirmed [19].

Concerning MRONJ development, only vague suppositions can be made. The most recognized and accepted theories concerning MRONJ development include—among others—an overall reduced bone-remodeling, a strong influence on oral soft-tissues, and the strong antiangiogenic potency of bisphosphonates and other drugs [20,21,22,23]. To our knowledge, an influence of methamphetamine on bone-remodeling is not known. Concerning the influence on soft-tissues, Haskin et al., reported extended, non-healing, necrotic skin wounds on the legs after long-time desomorphine abuse [24]. Baquero Escribano et al., described also that desomorphin is able to induce skin wounds and skin necrosis and further necrosis of blood and muscle tissues [25]. This might lead to the assumption that methamphetamine-induced osteonecrosis of the jaws might be caused or triggered by methamphetamine’s influence on soft-tissues and vascularization. However, another interesting hypothesis has been put forth. Rustemeyer et al., published a case report of a patient with an extended osteonecrosis of the maxilla after self-extraction of teeth and chronic methamphetamine abuse for 20 years. The authors concluded that (I) the self-extraction of the teeth in combination with bacterial wound contamination and (II) the inhalation of toxic phosphorus vapor, after heating white phosphor, might be responsible for ONJ development is this patient [26]. Hypothesis (II) is very interesting and might be a very relevant aspect since phosphor is an essential substitute for the illegal manufacture of “meth”. In this context, the phenomenon of the so-called “phossy jaw”—published by the British Medical Journal in 1899—was a horrible side effect for workers in the matchbox factories in the U.S. and in Great Britain at the end of the 19th century. They inhaled toxic phosphor steam from the matchbox production similar to “meth” users who smoke it and therefore also inhale toxic phosphor steam. The workers developed non-healing wounds, exposed jaw bone, and extraoral fistula in close correlation to the time they worked in the factory and were directly exposed to the steam [27].

Besides pathophysiology, the prevention and treatment of patients with “meth mouth” and methamphetamin-induced osteonecrosis of the jaws is relevant. Rommel et al., performed a systematic study and analyzed the effects of accompanying factors and circumstances on oral health in 100 patients with chronic methamphetamine abuse, such as—among others—the socioeconomic status of the patients and details of their methamphetamine consumption. The findings of this study demonstrated that “meth” abuse is more frequent in patients with unstable social circumstances. Further, significantly reduced oral and dental care has been found in the methamphetamine group compared to the controls [28]. In a following study, Rommel et al., analyzed the influence of methamphetamine abuse on teeth and intraoral tissues in 100 patients. The results of this study demonstrated a significantly increased number of carious lesions and periodontitis in combination with an overall reduced oral care and hygiene in patients with methamphetamine abuse. The authors also described the necessity of specific prevention and treatment concepts for patients with methamphetamine abuse [29]. Hamamoto and Rhodus completed a review and reported that finishing the drug abuse represents the most relevant aspect for prevention and treatment of oral methamphetamine side effects [30]. This recognition has also confirmed by other authors. Wang et al., reported that the cessation of “meth” abuse is the most relevant aspect. Further, simplified treatment plans and procedures, short treatment times, and strict postoperative care are relevant factors [31]. In another study, it has been demonstrated that a significantly relevant association between the number of decayed teeth and the duration of “meth” abuse exists. Four years of “meth” abuse has been found to be a critical time period for an increased risk of “meth mouth” development [32]. Concerning the prevention and therapy of methamphetamine-induced osteonecrosis of the jaws, non-surgical (e.g., mouth rinses) and surgical treatment protocols (e.g., necrosis excision, osteotomy and plastic coverage), similar to MRONJ patients, might be possible. While some authors prefer the surgical therapy in MRONJ management, making any reliable statements as to whether it is advisable to do this with patients with methamphetamine abuse is not yet possible and should be evaluated in further clinical trials [33].

Methamphetamine-induced osteonecrosis of the jaws was most frequently reported in patients with desomorphin abuse. Compared to “crystal meth”, desomorphin is often injected intravenously. The term “Krokodil” for desomorphin describes the phenomenon of the green and scaly skin around the injection side. Poghosyan et al., designed a retrospective study in patients with jaw necrosis after desomorphin abuse. In total, 35 lesions were found in 29 mandibles and 21 lesions in 19 maxillas. The authors concluded that there are two main aspects for treatment success: (I) cessation of desomorphin use pre- and postoperatively and (II) the resection of the necrotic bone. Recurrence was observed in nearly 25% of the cases, exclusively in the mandible [7]. Further, Hakobyan and Poghosyan reported a case of desomorphin-induced osteonecrosis of both jaws after 18 months of desomorphin abuse and 8 months of desomorphin withdrawal prior to surgery. In a follow-up period of 36 months, no recurrence of jaw- of soft-tissue necrosis was observed [8]. Faucette et al., reported sinusitis and mucoceles of the maxillary sinus in two patients with long-term methamphetamine abuse, probably with odontogenic origin [34].

To conclude, methamphetamine abuse as well as the resulting side effects of “meth mouth” and methamphetamine-induced osteonecrosis of the jaws as a special entity of MRONJ might become more relevant in the field of dentistry and oral- and maxillofacial surgery. The exact correlations between methamphetamine abuse, “meth mouth”, and the development of jaw necrosis are still unclear and should be further investigated. To date, it might be useful to treat patients with chronic methamphetamine abuse just like MRONJ patients.
